# The role of rhBMP-2 in mandibular bone regeneration following tooth extraction through HIF-1α and VEGF-A expression: An Immunohistochemical study

**DOI:** 10.1016/j.jobcr.2025.02.001

**Published:** 2025-02-13

**Authors:** Christian Khoswanto, Ira Kusuma Dewi

**Affiliations:** a Department of Oral Biology Faculty of Dentistry, Airlangga University, Surabaya, Indonesia; b Dentistry Clinic Research, Surabaya, Indonesia

**Keywords:** rhBMP-2, VEGF-A, HIF-1α, Bone graft, Tooth socket

## Abstract

**Background:**

Dentists frequently conduct tooth extractions when there is damage to the tooth or the tissue that supports it. When a tooth is extracted, the alveolar bone will sustain injury. Most of the initial bone volume is lost following the healing phase after extraction. Rehabilitation must start as soon as a tooth is missing, mainly because the alveolar bone is severely damaged during the tooth extraction, particularly in the buccal region where the tooth is removed. Dental implant is one method of replacing lost teeth. One of the most important elements influencing the clinical result of dental implants is a change in the dimension of the alveolar bone. Several bone-grafting techniques, such as socket preservation techniques, have been developed to increase the volume of bone throughout the healing phase after tooth extraction. This study aims to assess the impact rhBMP-2 on creating bone regeneration through VEGF and HIF-1α in the mandibular socket post-extraction in Wistar rats.

**Method:**

On the anterior side of the mandible, in the socket area where the tooth was extracted, rhBMP-2 was injected into the socket, and the xenograft material was applied with a syringe. Male, 9-week-old Wistar rats were chosen (n = 30).

**Result:**

Our statistical evaluations have revealed a significantly higher VEGF-A and HIF-1α expression post-extraction of the rhBMP-2 and xenograft group compared to other group treatments. These findings are significant as they provide a deeper understanding of the mechanisms involved in bone regeneration post-extraction.

**Conclusion:**

Our study suggests that injecting rhBMP-2 into the grafted material and socket extraction during GBR dramatically increases the expression of VEGF-A and HIF-1α. These findings have the potential to significantly impact oral surgery and regenerative dentistry, opening up new possibilities for enhancing bone regeneration techniques.

## 1 Introduction

Dentists frequently conduct tooth extractions when there is damage to the tooth or the tissue that supports it. When a tooth is extracted, the alveolar bone will sustain injury. Most of the initial bone volume is lost following the healing phase after extraction. Rehabilitation must start as soon as a tooth is missing, mainly because the alveolar bone is severely damaged during the tooth extraction, particularly in the buccal region where the tooth is removed. One typical approach is the extraction socket-healing concept used to observe the process of bone tissue healing in dentistry. Three phases of IPR, the inflammatory (I), proliferative (P), and remodeling phases (R) of the alveolar bone wound healing process occur after tooth extraction and last for several months. Initially, blood is drawn into the extraction socket, and during the inflammatory phase, Platelets are pulled into the region of the wound. Initial Inflammatory responses start to clear the wound site once the blood clot (BC) closes, the bleeding stops, and the wound is covered. After that, angiogenesis starts, and a temporary connective tissue matrix develops within two weeks of extraction. Woven bone forms in the proliferative phase due to bone-forming cells and arteries entering the temporary matrix. In the first three months after extraction, more than sixty percent of the bone is rebuilt; however, this immature woven bone cannot support weight and is gradually replaced by mature lamellar bone and bone marrow over several months. Most of the initial bone volume is lost following the healing phase, particularly on the facial side. A change in dimension is one of the most important elements influencing the clinical result of dental implants. Several bone-grafting techniques, such as techniques for preserving sockets, have been developed to retain or increase the volume of bone throughout the healing phase. Developing a plan to promote bone development requires a thorough understanding of wound healing mechanisms at the cellular level because growth factors are dynamically regulated to draw the right cells into defects and stimulate bone creation. During inflammatory stages, platelets release PDGF to promote cell proliferation and chemotaxis, which is critical for wound healing. As inflammatory cells migrate into the wound site, pro-inflammatory cytokines such as IL-1, IL-6, and TNF-α are produced. The angiogenesis process is crucial for supplying cellular and nutritional support during proliferative periods. Hypoxia Inducible Factor 1-α (HIF-1α), VEGF-A, and PDGFs are the three factors that regulate angiogenesis. Osteogenesis and VEGF are intimately associated.^1^, ^2^, ^3^

The BMP-2 delivery method that is now in use is rhBMP-2 in protein form. Collagen sponges or bone substitutes are treated with rhBMP-2 by the defect morphology to promote bone regeneration through bone-grafting methods. Numerous clinical research on bone augmentation and alveolar bone regeneration have demonstrated the impact rhBMP-2 on these processes. However, the side effect associated with dosage is the most problematic aspect of rhBMP-2 therapy. Since BMP-2 is a late-acting growth factor, a high initial dose of rhBMP-2 is administered to defects to maintain an effective in vivo concentration throughout the healing period. The carrier system and the host's circumstances can also impact the correct dosages. The concentration of rhBMP-2 varied from 0.75 to 2.0 mg/mL in earlier research. Adverse consequences, including widespread swelling, the creation of seromas, and the formation of cystic bone lesions, have been linked to a physiological dose of rhBMP-2. There have been further studies on the use of rhBMP-2 that discuss things like the potential for bone outgrowth, interactions with exposed dura, immunogenicity, local toxicity, systemic toxicity, and osteoclastic activation.^4^, ^5^, ^6^, ^7^, ^8^

BMPs are a class of around 20 proteins in the TGF-β family. These glycoproteins stimulate Mesenchymal cells, which aid in their development into osteoblasts. Additionally, they control various biological processes, including fibroblast, endothelial, and osteoblast proliferation, migration, and differentiation. BMPs have a role in a variety of intracellular pathways that cross paths with proteins engaged in oncogenic pathways, which are responsible for the development of many cancers, including those that invade and colonize bone tissues. TGF-β superfamily has at least 15 different BMPs recognized as members. Some forms of recombinant BMPs, like recombinant human BMPs (rh-BMPs), have already been created. BMPs are regulatory genes; their sequences resemble those of TGF-β.^9^^,^^10^

Angiogenesis is shown in immunological responses, wound healing, and inflammatory reactions. Developing new venules and arterioles during tissue recovery is necessary for gingival healing. The percentage of VEGF-A isoform and Ki-67 in gingival soft tissue are two variables that could be used to assess the impact of rh-BMP proliferation at the gingival level.^11^^,^^12^ This study aims to determine the effect of rhBMP-2 on the creation of bone regeneration through VEGF and HIF-1α expression in the mandibular socket post-extraction in Wistar rats.

## 2 Material and methods

### 2.1 Ethics statement. DSMATERIALS

The Animal Committees of the University Graduate Schools of Dentistry have approved this study. Every attempt was made to reduce the suffering of the animals during all animal investigations, which were carried out under sodium pentobarbital anesthesia and in compliance with the guidelines for animal studies. The ethical clearance certificate number is 411/HRECC.FODM/IV/2023.

### 2.2 Animals

9-week-old male Rats of the Wistar strain (n = 30) were used.

### 2.3 Alveolar socket Preparation

On the anterior side of the mandible, in the socket area where the tooth was extracted, Nothing was inserted into the socket. (Group 1). Bone xenograft material (Medpark, Korea, size 1,0 mm) was inserted for Group 2. Bone xenograft material (Medpark, Korea, size 1.0 mm) + 1 mL of rhBMP-2 0.25πg/mL (Cowell Medi R&D, Korea) was inserted for Group 3.

### 2.4 Application of material to the socket

Each socket is filled with one of the following.

Group 1: Control
Group 2: xenograft
Group 3: rhBMP-2 + xenograft

Ten samples were used for each series of materials.

#### 2.4.1 Immunohistochemical analysis and Histological techniques

On the third day following tooth extraction, the expression of VEGF-A and HIF-1α was monitored in this study. The tissue samples were sent to the histology lab after being fixed in ten percent buffered formalin. A total of two or three 4 μm-thick serial sections were made. After that, the sections were rehydrated and deparaffinized using progressively less amounts of alcohol and xylene. For two to 5 min, Using 10 mM citrate buffer (pH 6.0), antigen retrieval was carried out in a pressure. After using 3 % hydrogen peroxide for 15 min to suppress endogenous peroxidase activity, All slides were incubated for 4 h at the standard temperature using an optimum dilution of 6 μg/mL of primary anti-VEGF and HIF-1a rabbit polyclonal Ab (Abcam Inc., USA). Following additional incubation for 45 min with the secondary antibody and 30 min with streptavidin peroxidase, visualization was carried out for 10 min using newly made diaminobenzidine (DAB) chromogen. Lastly, the staining VEGF-A expression was investigated by looking at the slides under a microscope. Every group's slide had five fields that were seen at 100× magnification and utilized to count and identify VEGF-A and HIF-1α expressions.

#### 2.4.2 Statistical analyses

The statistical software SPSS 26.0 was used for the analyses. A statistically significant p-value for the analysis was defined as less than 0.001. The significant finding (p < 0.001) led to the proposal of a 95 % confidence level. For each of the three groups—group 1 (control), group 2 (xenograft), and group 3 (with rhBMP-2 and xenograft) The VEGF-A and HIF-1a counts were calculated and their standard deviations were computed. Additionally, The Shapiro-Wilk and Kolmogorov-Smirnov tests for normality were used to determine whether the variables had a normally distributed distribution. Oneway ANOVA was used to determine the significance of the data changes in the rats' mandibular bone between the treatment and control groups see (Fig. 2).

**Fig. 1:**
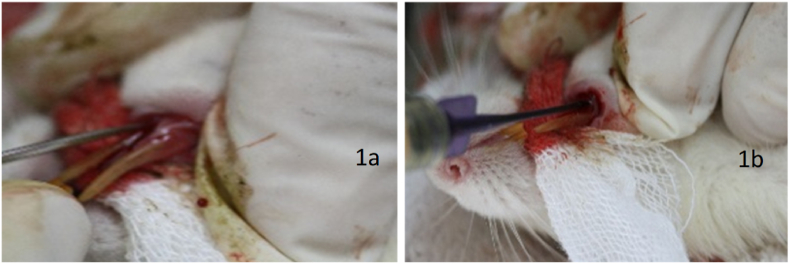
The procedure for removing a Wistar rat's anterior teeth is shown in Fig. (1); research material is inserted into the tooth socket following extraction (1b).

**Fig. 2:**
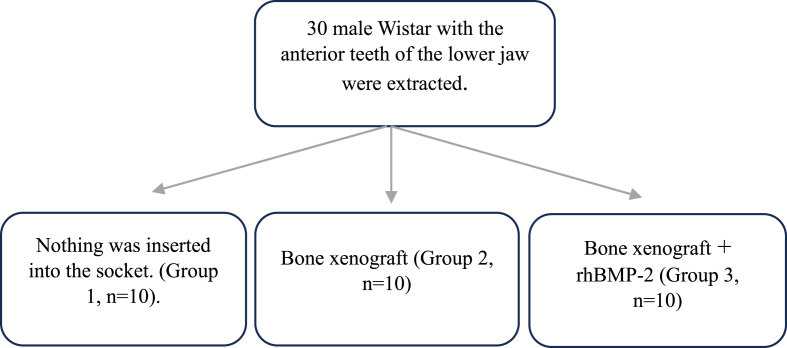
Methodology study design.

## 3 Results

### 3.1 Control group

**Fig. 3:**
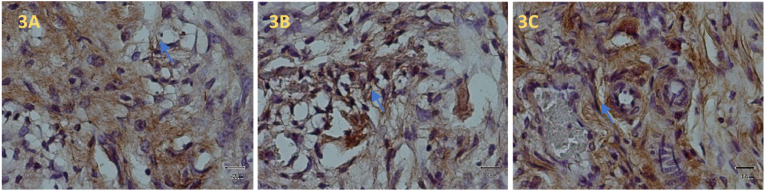
VEGF-A expression was visible in the wound area on day 3. Control Group (3A), Xenograft (3B) and rh BMP-2 + Xenograft (3C).

**Fig. 4:**
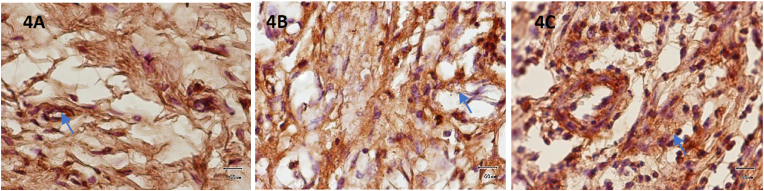
HIF-1α expression was visible in the wound area on day 3. Control Group (4A), Xenograft (4B) and rh BMP-2 + Xenograft (4C).

Following assessment on the third day, the region surrounding the wound showed signs of VEGF-A expression (Fig. 3A). Around the control group, there was a noticeable inflammatory reaction, including neutrophil infiltration. Compared to inflammatory cells, endothelial cells do not appear to express HIF-1α in greater quantities during the development of new blood vessels (Fig. 4A).

### 3.2 Xenograft

New blood vessels were formed, indicating more VEGF-A expression in the xenograft application group exclusively on tooth extraction wounds area (Fig. 3B). In this group, HIF-1α significantly increased expression compared to the control (Fig. 4B).

### 3.3 rhBMP-2 + xenograft

Compared to the previous two groups, the rhBMP-2 + Xenograft group applied to the tooth extraction wound socket on the third day demonstrated a significant increase in VEGF-A (Fig. 3C). The same result was also shown in HIF-1α expression (Fig. 4C)

Table 1. Displays the average VEGF-A and HIF-1α expression in Wistar rats after extraction. On the third day of the rhBMP-2 + xenograft group, the light microscopy view of the socket following tooth extraction reveals increased significantly VEGF and HIF-1α expression in comparison to the other group. A Kolmogorof-Smirnov test was performed on the data to confirm the normality of the distribution. A substantial difference in treatment between each group is revealed by the ANOVA test and LSD test (p = 0.001).

**Table 1:** shows the mean and standard deviation of VEGF-A & HIF-1α expression in the treatment and control groups.

Group	X±SD Day 3 VEGF-A	X±SD Day 3 HIF-1α
**K**	14.20^a^ ± 1.47	13.20^a^ ± 1.22
**Xenograft**	18.10^b^ ± 1.44	16.60^b^ ± 1.43
**RhBMP-2 + Xenograft**	24.00^c^ ± 1.76	20.40^c^ ± 1.77

Note that the varied superscripts revealed a significant difference (0.05).

## 4 Discussion

Numerous investigations have shown that rhBMP-2 can express HIF-1α in bone. However, no prior research has examined the possibility of a direct relationship between gingival vascularization enhancement and rhBMP-2.

Wounds caused by tooth extraction will damage the soft and hard tissue of the alveolar bone, damage blood vessels, and disrupt smooth blood flow. The lack of blood supply causes cell and tissue hypoxia, a lack of oxygen to meet the metabolic needs of cells and tissues. The state of hypoxia in cells and tissues, due to damage to blood vessels that provide oxygen and nutrients, will continue because the infiltration of inflammatory cells on days 2–3 also requires oxygen consumption. Oxygen is a significant component in determining the success of wound healing because processes such as cell proliferation, angiogenesis, and collagen synthesis require oxygen. Generally, oxygen in almost all tissues ranges from 2 to 9 %. Hypoxic conditions and decreased oxygen pressure typically occur in the wound area due to the unavailability of the required oxygen supply. Therefore, the availability and stability of the HIF-1α protein are needed to help the tissue repair process after tooth extraction wounds, considering that this protein is a controller of the response to hypoxic conditions in the tissue.^13^, ^14^, ^15^

Hypoxia in cells and tissues around the alveolar bone occurs due to an imbalance between available and needed oxygen. This occurs due to damage to the micro-blood vessels around the wound site. In the wound healing process, in the early stages of tooth extraction wounds, an inflammatory phase lasts 2–3 days. Damage to blood vessels in this phase will cause a lack of oxygen to cells and tissues, and along with the tissue repair process with an increase in inflammatory cells, it will cause an increase in the need for oxygen in these cells, which also increases the decrease in oxygen pressure. HIF-1α is a protein released by endothelial cells, osteoblast progenitors (osteoblast lining cells), mesenchymal cells, fibroblasts, and macrophages. HIF-1α and VEGF-A are two factors that play a significant role in regulating angiogenesis and forming bone repair. The interaction of HIF-1α and VEGF-A stimulates the initial formation of angiogenesis-osteogenesis, which forms bone growth and regeneration.^16^^,^^17^

Sampling in this study was done on the third day because VEGF-A levels peaked three days after the injury, and their baseline expression resumed by day six. Similarly, HIF-1α expression peaked on day three, when certain cells were actively differentiating, migrating, and proliferating, and then declined on day five.^18^^,^^19^

**Fig. 5:**
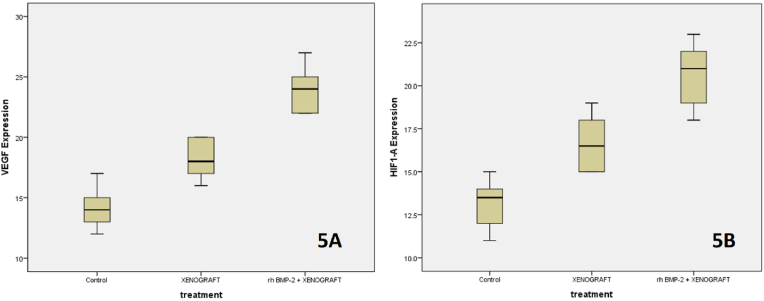
A boxplot diagram representing the data for each treatment group compared to the control group on day three is displayed as VEGF-A (5A) and HIF-1α (5B).

HIF-1α expression in the alveolar socket on day 3 after rhBMP-2 administration showed a higher increase in HIF-1α levels compared to the control group (Fig. 5). The increase in HIF-1α protein levels indicates that in the post-tooth extraction area, the decreased oxygen supply that disrupts cell survival can be overcome through an increased HIF-1α response, considering that the function of this protein is as a regulator of the response of cells that lack oxygen, can regulate oxygen homeostasis and activate the angiogenesis and osteogenesis processes through the activation of VEGF-A and BMP-2.^15^, ^16^, ^17^

Freitas et al. demonstrated in a groundbreaking study that while untreated sites lost alveolar ridge height, rhBMP-2 combined with collagen sponge preserved it at extraction socket sites. The effectiveness of BMP-7 and modified BM-MSCs for periodontal regeneration was validated by Jung et al.'s evaluation of the influence of BM-MSCs on periodontal tissue regeneration. Clinical observations of patients requiring extraction socket augmentation revealed a statistically significant difference in the efficacy of the 0.75 mg/mL and 1.50 mg/mL rhBMP-2 dosages. Insufficient bone development was observed at the extraction socket in 25 percent of implant area in the 0.75 mg/mL group and 56.25 percent of implant sites in the 1.50 mg/mL group.^20^^,^^21^

According to Scalzone et al., the test group (used rhBMP-2 during implant insertion) showed a notably higher rate of new vessel formation and higher VEGF-A marker in eighty percent of the samples compared to both control groups. These findings verify that the new gingival vascularization was augmented by rhBMP-2 injection. When it came to the height and volume of the bone graft, patients with cleft lip and palate who had maxillary alveolar restoration with or without rhBMP-2 graft showed similar results, despite a shorter period of hospital stay. Furthermore, BMP-2 promoted angiogenesis by upregulating VEGF-A activity, according to Kalay, E. et al. An essential process for the beginning and maintenance of new bone growth is angiogenesis. In gingival samples used in our investigation, BMP-2 injection accelerated the development of new blood vessels.^22^^,^^23^

Tooth extractions will undergo a healing process involving both the mouth cavity's hard and soft tissues. Gingival tissue is soft tissue, and alveolar bone is hard tissue. Angiogenesis and vasculogenesis, which restore blood flow to the injured tissue, fibroplasia, the development of fibrous tissue that aids in the development of granulation tissue, keratinocyte migration and proliferation-driven re-epithelialization, and myofibroblast-mediated wound contraction are the processes that make up wound healing. Angiogenesis and vasculogenesis are the processes by which new blood vessels form in healing tissue, re-establishing the vasculature required to carry oxygen and nutrients to the damaged tissue. Hypoxia triggers the activation of many angiogenic growth factor genes, such as VEGF-A, by building up HIF-1α.^24^ To our knowledge, nevertheless, no prior research has made an effort to link rhBMP-2 to an improvement in gingival vascularization through VEGF-A and HIF-1α. The rhBMP-2 combination with xenograft showed indicated a significantly greater creation of new blood vessels than in the other groups.

VEGF-A and rhBMP-2 work in concert to activate osteogenic and angiogenic processes, which have better healing qualities. Utilizing chemically altered mRNAs in conjunction with biomaterials represents another viable strategy for the management of bone deformities and injuries. Bone tissue regeneration may be considerably enhanced by the effective use of bioactive substances like BMPs. The optimal dosage and kinetic release profile, as well as the combination of compounds that works best in a given scenario, are still up for debate. Alveolar ridge preservation utilizing rhBMP2 and delivery techniques is a good treatment for implant insertion in the future. BMP and barrier membrane together may enhance periodontal tissue regeneration in clinical settings or possibly act as a bone substitute for regeneration.^25^

Numerous research investigations that were published emphasized the impact of BMPs on inflammation and periodontal repair. With the use of rhBMP-2, the study showed bone healing and management of tissue regeneration. Regretfully, there is a lack of information in the literature about how rh-BMP-2 directly affects the development of new arteriole and venule vessels in gingival tissue. VEGF-A and HIF-1α function as homing factors, stimulating MSC development into endothelial and osteogenic cells, according to Zhang et al.'s research. In vivo, VEGF-A and BMP-2 were released via porous silk scaffolds, which also facilitated the repair of bone defects by stimulating angiogenesis and the production of new bone. These results imply that VEGF-A and BMP combination therapy may be a viable approach for bone tissue regeneration.^26^^,^^27^

VEGF-A transfection produced more vessels than in the VEGF free circumstances, the combination of rhBMP-2 and xenograft dramatically enhanced bone growth. Thus, by modulating angiogenesis, VEGF-A expression may improve bone production. It is still unclear how rh BMP-2 affects vascular development and function, however, data that are now available suggest that BMP signaling is complicated and affects the vascular system. The control of the cell junctions that connect the endothelium cell system is essential for vascular development.^27^

Blood vessel production in the gingival tissue is influenced by rhBMP-2, This could affect the outcome of dental implants, periodontal regeneration, or wound healing. The treatment of injuries unique to particular organs, such as stroke recovery or cardiac regeneration, where angiogenesis is critical, may be affected more broadly by these discoveries. Examining how rhBMP-2 affects the development of new arterioles in various organs and gingival tissue demonstrates how adaptable rhBMP-2 is in controlling angiogenesis in various biological circumstances. These studies' differing therapeutic consequences and difficulties highlight the significance of conducting customized research for various medical and dentistry purposes.^28^^,^^29^

Research and clinical applications could benefit greatly from an examination of the impact of rhBMP-2 on the onset of new vascularization in gingival soft tissue. In many physiological and pathological situations, neovascularization is essential, especially for tissue regeneration and wound healing. For two reasons, rhBMP-2 affects this process in gingival tissue and may have significant effects on periodontal and dental therapies in addition to regenerative medicine applications. First, it might clarify the processes that underlie gingival tissue neovascularization, offering important new understandings of the many molecular pathways. Second, it might open the door to the creation of tailored treatments that use rhBMP-2 to promote the growth of new venule and arteriole in the gingival area. This would help treat gum disorders, increase implant success, and quicken the healing process following surgery.^30^^,^^31^ The results of this study will be useful for faster healing of the alveolar bone, because with the addition of rh BMP-2 angiogenesis and proliferation of endothelial cells in the socket area are formed more quickly, a significant increase in VEGF-A and HIF-1α expression proves this.

Future research is needed to explore the molecular pathways that underlie rhBMP-2's function in gingival angiogenesis, ascertain the ideal dose of rhBMP-2 administration to stimulate the new vessels, assess the effectiveness of combination therapies, and consider the possibility of rhBMP-2 working in concert with other growth factors to produce synergistic effects.

Our study's limitation is that the optimal dose of rhBMP-2 and its effects with Wistar age were not well studied. Longer-term studies are required to see how wound healing variables contributing to alveolar bone healing manifest. More investigation into these characteristics is necessary to fully comprehend the effectiveness of rhBMP-2.

## 5 Conclusions

Our findings showed that rhBMP-2 into the grafted material and socket extraction during GBR dramatically increases the expression of VEGF-A and HIF-1α, which in turn promotes the creation of neovascularization and may speed up the healing process of grafted areas.

## Author Statement

We wish to submit an article entitled: **The Role of rhBMP-2 in Mandibular Bone Regeneration Following Tooth Extraction: Immunohistochemical Study**, for consideration by Journal of Oral Biology and Craniofacial Research. I can confirm that the attached article is an original piece of work and that I am not submitting it to other journals for consideration. In this study, there were no funding sources involved in financial support for the conduct of the research.

## Funding

This research received no external funding

## Declaration of competing interest

The authors declare that they have no known competing financial interests or personal relationships that could have appeared to influence the work reported in this paper.
